# Prostate Adenocarcinoma with Signet-Ring Cells and Features of Mucin: A Clinical Case and Literature Review

**DOI:** 10.3390/medicina60060877

**Published:** 2024-05-27

**Authors:** Migle Sakalauskaite, Ausra Garnelyte, Ignas Civilka, Audrius Dulskas, Marius Kincius, Ausvydas Patasius

**Affiliations:** 1 Faculty of Medicine, Medical Academy, Lithuanian University of Health Sciences, 44307 Kaunas, Lithuania; 2 Department of Oncourology, National Cancer Institute, 08406 Vilnius, Lithuania; marius.kincius@nvi.lt (M.K.); ausvydas.patasius@nvi.lt (A.P.); 3 National Center of Pathology, Vilnius University Hospital Santaros Clinics, 08661 Vilnius, Lithuania; ausra.garnelyte@gmail.com; 4 Department of Abdominal and General Surgery and Oncology, National Cancer Institute, 08406 Vilnius, Lithuania; ignas.civilka@gmail.com (I.C.); audrius.dulskas@nvi.lt (A.D.); 5 Department of Public Health, Institute of Health Sciences, Faculty of Medicine, Vilnius University, 01513 Vilnius, Lithuania

**Keywords:** prostate cancer, signet-ring cell-like carcinoma, features of mucin

## Abstract

*Introduction*: Signet-ring cells are typically associated with mucin-secreting epithelium; thus, they are most commonly found in the gastrointestinal tract, but not exclusively. Primary signet-ring cell carcinoma of the prostate is a rare and poorly differentiated, aggressive acinar adenocarcinoma variant with a grim prognosis. *Clinical Case*: In June of 2023, a 54-year-old Caucasian male presented with a complaint of lower urinary tract obstructive symptoms with occasional macrohematuria, non-specific body aches, and shortness of breath. A prostate specimen obtained in transurethral resection of the prostate was sent for histopathological examination. After a series of extraprostatic diagnostic workups, including fibrogastroduodenoscopy, colonoscopy computed tomography imaging, and immunohistochemical studies, the patient was diagnosed with primary prostatic signet-ring cell adenocarcinoma stage IV. Unfortunately, due to the advanced stage of the disease, PE, and third-degree thrombocytopenia, the patient was not a candidate for chemotherapy and died of cardiopulmonary insufficiency later that week. *Discussion*: Prostatic signet-ring cell carcinoma accounts for 0.02% of all prostate adenocarcinoma cases. Due to its nature and epidemiology, a diligent extraprostatic investigation has to be carried out. The disease often presents with unremarkable clinical symptoms and variable serum prostate-specific antigen results, which may contribute to its late diagnosis. Inconsistent immunohistochemical findings and an unpredictable response to hormonal treatment together pose both diagnostic and therapeutic challenges that negatively affect the prognosis. *Conclusions*: This study highlights the importance of a multidisciplinary approach and the need for diagnostic and therapeutic consensus within the research community in search of the primary site of the disease, which may positively influence the prognosis.

## 1. Introduction

Prostate cancer is the second most commonly diagnosed cancer in men, with a rapidly growing age-related incidence and mortality each year [1]. The majority of prostate cancers are not clinically evident and are of a relatively low virulence. This may be true in the case of an acinar adenocarcinoma, which makes up 93% of all prostate cancer cases [2]. Even though the acinar type is the most common, both the signet-ring cell subtype and mucinous histological pattern are considered to be extremely rare [3,4].

Signet-ring cells acquire their histological appearance in the presence of an intracellular clear cytoplasmic vacuole, which pushes the nucleus into the periphery, giving it a crescent shape [5]. The cells were first grossly characterized by their distinctive looks and diffuse submucosal growth pattern in the early 1950s [6]. The variant is predominantly observed in the gastrointestinal tract, emphasizing the stomach and colon; therefore, these organs are the first ones to be ruled out in suspicion of metastatic disease and are a reference point in other-organ signet-ring cell carcinoma (SRCC) cases [5]. However, it is not an uncomplicated process since early gastric SRCC is nearly macroscopically invisible, meaning that in most cases, it is diagnosed rather late, posing a great diagnostic and therapeutic challenge [7].

Primary prostatic signet-ring cell-like adenocarcinoma (PPSRCA) is an exceptionally rare, poorly differentiated epithelial cancer with unremarkable genitourinary complaints, inconsistent immunohistochemical study findings, non-universally followed classification criteria, and unestablished diagnostic and therapeutic protocols. Altogether, this negatively influences the already poor 5-year survival rate [5,6,8].

To gain a more comprehensive understanding of the entity, we herein present a clinical case of prostate adenocarcinoma with signet-ring cells and features of mucin observed in a single tertiary cancer center in Lithuania.

## 2. Case Report

**Figure 1:**
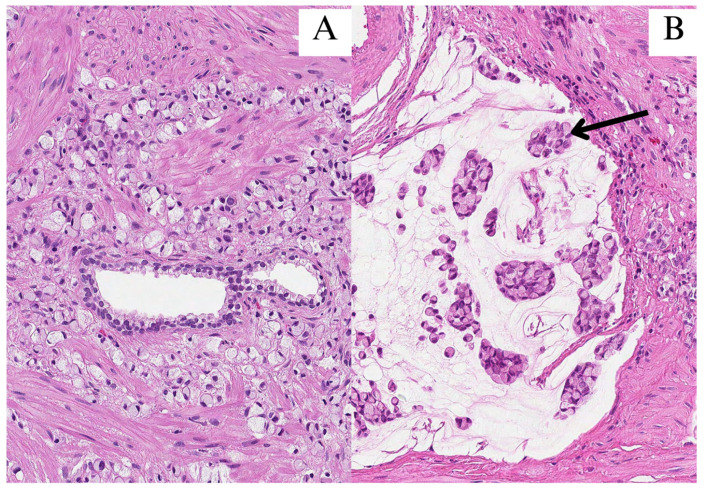
All images were stained with hematoxylin and eosin (HE) and 200× magnification. (**A**) Signet-ring cells infiltrated in between prostate glands; (**B**) clusters of signet-ring cells (arrow) in pools of extracellular mucin.

**Figure 2:**
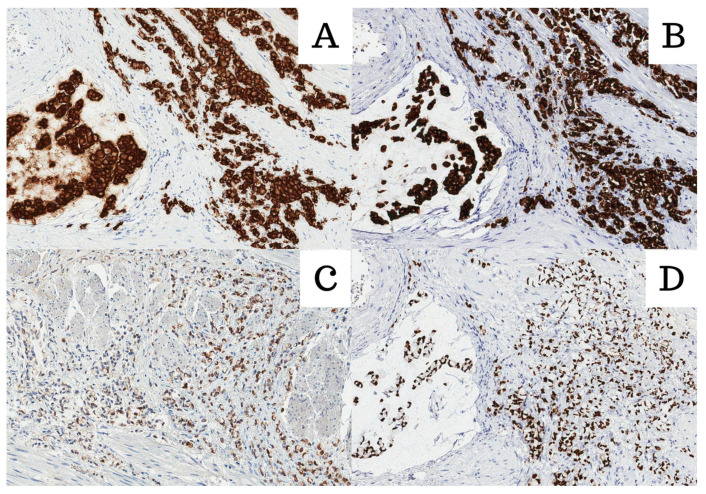
All images were stained with HE and 200× magnification. (**A**) Cadherin 17; (**B**) Muc2; (**C**) CK20; (**D**) CDX2.

In June of 2023, a 54-year-old Caucasian male with an unremarkable medical history presented with symptoms of episodic hematuria, severe non-specific body aches, shortness of breath, and anuria, which was initially treated with cystostomy before undergoing transurethral prostate resection (TURP). Histopathological examination of the resection revealed a mass of poorly differentiated (G3) adenocarcinoma of an unknown primary site with a diffuse distribution of signet-ring cells (40%) with intracellular and extracellular mucin that constituted 20% of the tumor found within the specimen (Figure 1). An immunophenotype was later determined, and the tumor cells were found to be positive for CDX2; cadherin 17; MUC2; focally for cytokeratin 20 (CK20) and synaptophysin; and negative for CK7, NKX3.1, GATA3, SATB2, MUC5, and MUC6 (Figure 2). Both the visual representation and immunophenotype were suggestive of a metastatic tumor of the lower gastrointestinal tract. Thus, further investigation to verify the primary site was necessary. In search of a primary site, fibrogastroduodenoscopy, chest and abdominal computed tomography (CT), and lesser pelvic magnetic nuclear resonance (MRI) scans were performed, subsequently disclosing erosive gastroduodenopathy, pulmonary embolism (PE), direct seminal vesicle and urinary bladder infiltration, and multiple osteosclerotic metastases—which led to the conclusion that the primary site of SRCC was the prostate gland itself. A TNM class and stage were assigned accordingly—cT4N1M1c stage IV. The patient, with such an advanced disease, did not meet the criteria for radical prostatectomy (RP) or radiation therapy (RT). He was denied chemotherapy due to third-degree thrombocytopenia, due to which antithrombotic treatment, which first was prescribed for PE treatment, was discontinued as well. Infusions of zoledronic acid were initiated. The patient died of cardiopulmonary insufficiency later that week—27 days after the diagnosis was made.

## 3. Literature Review

The literature review was performed on PubMed using the search words “primary signet ring cell carcinoma”, excluding prostate-non-related cases. Some authors could not determine the primary site of the disease. However, since the tumor was found within the prostate, the studies were not excluded from the literature review for comparative purposes. Studies with a history of other organ-confined cancers, except that of genitourinary or gastrointestinal tracts, were excluded.

The selected clinical cases were sorted into four categories according to the stage of the disease at the time of presentation: localized, advanced, distant, or unknown [9]. Other available information was collected to determine the presence of possible trends or patterns among the patients: age, main complaints, serum prostate-specific antigen (sPSA), and used immunohistochemical markers.

Out of 23 analyzed primary prostatic SRCC cases, 9 were found to be advanced, 9 were distant at the time of presentation, and 3 were localized (Table 1). The complaints often consisted of obstructive lower urinary tract symptoms, occasional gross hematuria, and symptoms that may be associated with distant metastasis. Findings including sPSA values or immunohistochemical staining were inconsistent, although most (16/23) were positive for PSA. One of the analyzed cases (case no. 5) that was immunohistochemically negative for PSA was later found to be a metastatic disease from the upper gastrointestinal tract and was not excluded from the study for comparison.

## 4. Discussion

Primary signet-ring cell adenocarcinoma of the prostate was first mentioned in the late 1970s. Since then, fewer than 100 cases have been published in English literature [33]. It is a rare, high-grade (Gleason grade 5) acinar adenocarcinoma subtype characterized by its distinctive intracellular substance-containing vacuole, which displaces the nucleus into the periphery of the cell, giving it a crescent shape [10,34]. The content of the vacuole may vary [35]; however, according to the latest edition of Prostate and Urinary Tract Tumors classification by the World Health Organization, in cases where the vacuole contains mucin, the tumor should be named signet-ring cell-like adenocarcinoma rather than signet-ring cell adenocarcinoma. Furthermore, vacuolated cells are associated with a greater Gleason pattern, which independently worsens the prognosis [4].

The diagnostic criteria suggests that the diagnosis of signet-ring cell adenocarcinoma of the prostate should be assigned only when the vacuolated cells make up at least 25% of the entire tumor volume, which may be evaluated on the whole-organ specimens obtained in surgery and not biopsy. Otherwise, in the cases where the cellular volume requirement is not met, or the specimen is obtained in a biopsy, the entity should be referred to as a prostate adenocarcinoma with signet-ring cells instead [4,34,36]. The criteria of the cellular composition is not strictly followed since cases with less than 20% have been accepted as SRCC of the prostate [29]. In contrast, reference [11] claimed that any histological specimen obtained may be used for diagnostic purposes validating those that were taken in TURP. This may be useful in cases when patients present late in the course of the disease and do not meet the criteria for radical prostatectomy. However, artifacts—like lymphocytes or vacuolated smooth muscle cells—mimicking SRCC in TURP specimens are not uncommon [3]. Fortunately, the confusion may now be avoided by applying specific immunohistochemical studies, including leukocyte common antigen and alpha-smooth muscle actin [12].

Despite constantly changing terminology, the lack of strict diagnostic criteria, and unestablished investigative protocols, which all together pose additional issues calculating the incidence of the disease, it is clear that a true prostatic SRCC is extremely rare, with an estimated prevalence of 0.02% among all prostate adenocarcinoma cases [5]. Due to its rare nature, the close epidemiological relation to the gastrointestinal tract, and diffuse, lateral, submucosal growth pattern, a diligent diagnostic workup for differentials must be carried out since the location of the primary tumor may be an independent factor for the cause-related survival and virulence of the disease [7,13]. The investigations should include upper gastric endoscopy, colonoscopy, cystoscopy, and abdominal computed tomography to exclude metastatic involvement of the prostate [10]. It may not be a routine procedure, yet some patients may benefit from random gastric biopsies [14]. Clearly, the method is not the most reliable for obvious reasons, yet it is important to recognize that early gastric SRCC may not be macroscopically visible, and late gastric SRCC may occasionally appear as mucosal erosions [7].

In addition to the gastrointestinal tract, particular attention must be paid to exclude SRCC of organs in close anatomical proximity to the prostate, like the urinary bladder or rectum [15]. These organs should not stain for PSA but may be strongly positive for prostate-specific acid phosphatase (PSAP) on the immunohistochemical study perhaps due to shared cloacal derivation [37].

Even though PSA is considered to be a highly specific marker for prostate tissue, its expression was found to be lost in poorly differentiated cells. This may pose an additional diagnostic struggle in differentiating between primary and metastatic disease [16,17,38]. It is worth noting that NKX3.1 may increase PSA sensitivity when applied in combination [39]. However, before NKX3.1 stain was available, it was speculated that SRCC of the prostate could be classified into two types: tumors that react positively to PSA and simultaneously negatively to carcinoembryonic antigen (CEA) and those that do not react to PSA yet express positivity in reaction to CEA [18]. The possibility of the variants has not been disproved and may be highly significant in choosing the most appropriate therapeutic approach.

Some studies claim they could not determine the primary site of the disease. This is true in up to 5% of all metastatic disease cases, even though immunohistochemical studies were performed [8,40]. Interestingly, [19] reported an alternative approach to the problem, which yielded great results in the case of T3b primary prostatic SRCC by applying colorectal SRCC cancer treatment based on the immunohistochemical study findings of the prostate biopsy alone. The study implies that the treatment may be applied based on the histological and molecular aspects of the disease rather than following organ-oriented treatment protocols.

SRCC of the prostate is often described as having an aggressive clinical course and unpredictable response to hormonal therapy. Some publications argue that this presumption may have arisen from the fact that most patients were diagnosed at late stages of the disease, before the serum PSA marker era, and if diagnosed early, the variant is of similar prognosis to the usual acinar prostate adenocarcinoma (PA) [10,20,35]. Unfortunately, other articles similar to our literature review show that serum PSA values greatly vary and are thus not entirely reliable in the diagnosis of prostate SRCC [21,41]. This may play a role in late diagnosis. In addition to this, non-specific clinical symptoms, often including lower urinary tract obstruction and occasional gross hematuria, may be other factors greatly contributing to the late diagnosis.

Although we lack well-established guidelines, the current treatment of prostatic SRCC is rather similar to that of the traditional adenocarcinomas of the prostate—which includes a variable combination of surgical procedures, hormonal therapy, adjuvant radiotherapy, and/or chemotherapy [10]. Even though the multimodal aggressive approach is very reasonable, a combination of radiotherapy and hormonal therapy may be an appropriate alternative therapy for prostate SRCC treatment [12].

Similarly to signet-ring cell prostate cancer, mucinous (i.e., colloid) carcinoma (MC) is another rare variant of the usual acinar PA. Since one-third of all prostate adenocarcinomas contain some focal differentiation of mucin, it is important to stress that MC is characterized by the extracellular pools of mucin. This must occupy at least 25% of the entire tumor volume on a whole organ specimen; otherwise, such tumors are described as “with features of mucin” [36]. It is graded on the structural architecture of the histological view, irrespective of the mucinous component. Similarly to the usual PA, it is associated with elevated serum PSA levels before diagnosis and has a comparable response to treatment and prognosis [42].

In exceptionally rare cases, MC may contain signet-ring cells, making the entity known as mucinous carcinoma with signet-ring cells (MCSRC). The two variants need to be distinguished from each other since the presence of SRC seems to tremendously worsen the prognosis, with a 5-year survival rate equal to zero [21,38,43].

## 5. Strengths and Limitations of the Study

To our knowledge, this is the first documented case of signet ring cell adenocarcinoma of the prostate in Lithuania. The clinical case is of great importance for the scientific community and practitioners, serving as a major educational tool for raising awareness and choosing appropriate diagnostic approaches in rare cases of prostate cancer. It has the potential to improve overall outcomes by bringing awareness and aiding in choosing an appropriate diagnostic approach in rare cases of prostate cancer.

Besides the strengths, the study has some limitations. We do not have enough clinical data to prove that the prostate is the primary site of the SRCC, given that we lack data on specific immunohistochemical stains. Additionally, the data that we have closely resemble that of the lower gastrointestinal tract. An autopsy was not performed. Furthermore, in retrospect, erosive gastropathy should have raised suspicion for gastric SRCC. Finally, minding the submucosal growth pattern and the virulence of small cluster cancers of SRCC, the CT imaging may not be sensitive enough to rule out the gastric SRCC diagnosis.

## 6. Conclusions

In summary, this clinical case highlights the importance of a multidisciplinary approach in conducting comprehensive diagnostic studies that unfortunately remain unstandardized to this day. The rarity of the entity together with the late diagnosis poses a great challenge in choosing an appropriate treatment to improve the prognosis.

## Figures and Tables

**Table 1:** The literature review of the 23 analyzed cases. N/A—not available.

No.	Reference	Age	sPSA (ng/mL)	Stage	Immunohistochemical Study
1	[10]	63	16.39	Advanced	Positive: PSA, P504S. Negative: CK7, CK20, SMA, LCA, PAS, Alcian blue.
2	[11]	61	14.7	Distant	Positive: PSA, P504S, Pan-CK, CD68. Negative: HMW-CK.
3	[12]	70	7.26	N/A	Positive: PSA, PSAP. Negative: LCA, ASMA.
4	[13]	70	27	Advanced	Positive: PSA, PA, Pancytokeratin. Negative: CK20, CK7, PAS, Alcian blue, Mucin. GI biopsy: negative for SRCC
5	[14]	67	4.33	Advanced *	Positive: CEA, CK20, LP34, Cam 5,2. Negative: PSA, CK7. GI biopsy—positive for SRCC
6	[15]	57	Normal	Advanced	Positive: PSA, PSAP, PAS, EMA, Alcian blue, CEA, mucicarmine. Negative: N/A
7	[16]	67	N/A	Distant	Positive: PAS, CK, Alcian blue, CEA, mucicarmine, weakly PSA. Negative: PSAP.
8	[17]	81	100	Advanced	Positive: PSA, PSAP, weakly PAS. Negative: Alcian blue, Mucicarmine, CK20, CK7, LCA, SMA, CEA.
9	[18]	61	Normal	Advanced	Positive: PAS, Muc, CA 19-9, CEA. Negative: PSA, PSAP.
10	[19]	65	6.6	Advanced	Positive: PSA, CK20, Alcian blue, Acid-Schiff. Negative: CK5, CK6. GI biopsy: negative for SRCC.
11	[20]	85	9.1	Localized	Positive: PSA. Negative: CK7, CK20.
12	[21]	47	0.117	Advanced	Positive: PAS, Alcian blue, CEA. Negative: PSA.
13	[22]	72	6.5	Localized	Positive: PSA, AMACR, Pancytokeratin. Negative: PAS, Mucicarmine, Alcian blue, CK7, CK20, LCA, SMA, CEA.
14	[23]	66	>6658	Distant	Positive: PSA, PAS, PSP. Negative: N/A
15	[24]	70	N/A	N/A	Positive: PSA, PSAP, PAS, Alcian blue, Mucicarmine. Negative: N/A
16	[25]	65	1990	Advanced	Positive: PSA, P504S. Negative: CK20, Mucin.
17	[26]	74	10.3	Distant	Positive: N/A Negative: N/A
18	[27]	72	470	Advanced	Positive: PSA. Negative: PAS, Alcian blue, Mucicarmine.
19	[28]	70	N/A	Distant	Positive: PSA, PSAP. Negative: Mucicarmine, mucopolysaccharide, PAS, Alcian blue.
20	[29]	65	151	Distant	Positive: EMA, Anti-PSAP. Negative: N/A
21	[30]	76	237	Localized	Positive: PSA. Negative: CEA.
22	[31]	65	Normal	Advanced	Positive: PSA. Negative: PAS, Alcian blue, p53, CEA.
23	[32]	56	0.64	Advanced	Positive: Cyclin D1, EGFR, P53, CK20, CX-2. Negative: Bcl2, c-erbB2, AMACR, CK7, TTF-1.

## Data Availability

All data generated or analyzed during this study are included in this published article.
